# Supplementary material for: Cuticle supplementation and nitrogen recycling by a dual bacterial symbiosis in a family of xylophagous beetles
Source: ISME J. 2023 Apr 21;17(7):1029–39. doi: 10.1038/s41396-023-01415-y (PMC10284843; doi:10.1038/s41396-023-01415-y)
Supplement: Supplementary file 1 — Supplemental material [file 41396_2023_1415_MOESM1_ESM.pdf]

Supplementary material: Cuticle supplementation and nitrogen recycling by a dual bacterial symbiosis in a family of xylophagous beetles

Julian Simon Thilo Kiefer[1], Eugen Bauer[1], Genta Okude[2,3], Takema Fukatsu[2,3,4], Martin Kaltenpoth[1,5], Tobias Engl[1,5,*]

[1]Department of Evolutionary Ecology, Institute of Organismic and Molecular Evolution, Johannes Gutenberg-University, Mainz, Germany

[2]Bioproduction Research Institute, National Institute of Advanced Industrial Science and Technology, Tsukuba 305-8566, Japan

[3]Department of Biological Sciences, Graduate School of Science, University of Tokyo, Tokyo 113-0033, Japan

[4]Graduate School of Life and Environmental Sciences, University of Tsukuba, Tsukuba 305-8571, Japan

[5]Department of Insect Symbiosis, Max-Planck-Institute for Chemical Ecology, Jena, Germany

[*]corresponding author: tengl@ice.mpg.de

Competing interests

The authors declare no competing interests.

Supplementary tables & figures

Table S1: Sampling information of collected Bostrichidae specimens. JKI = Julius Kühn-Institute / Federal Research Centre for Cultivated Plants; BAM = Federal Institute for Materials Research and Testing.

- Available as separate Excel file -

Table S2: General features of the symbiont genomes based on annotations with Prokka. LSU: Large subunit ribosomal protein, SSU: Small subunit ribosomal proteins.

- Available as separate Excel file -

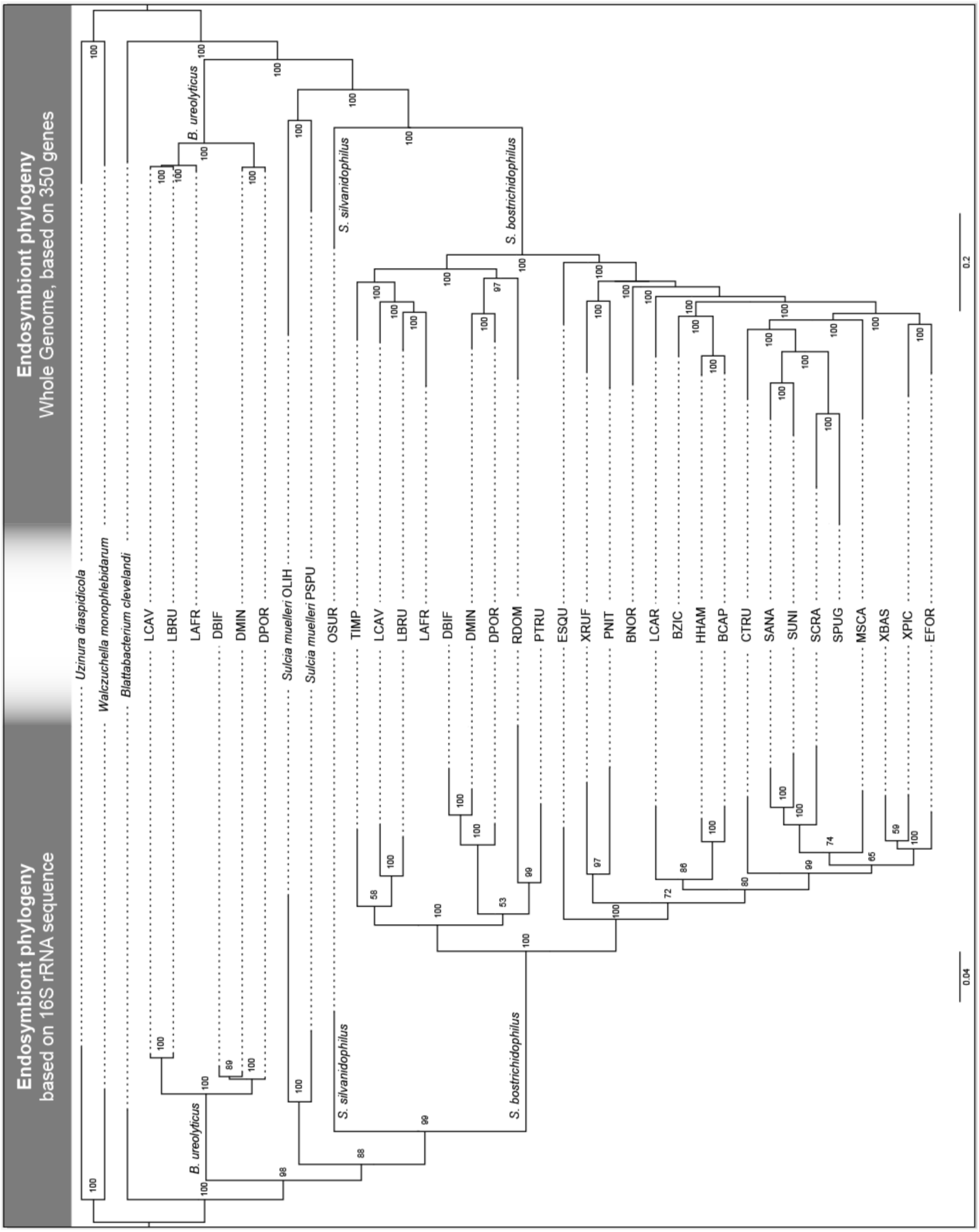

Figure S1: Comparison of Bacteroidota symbiont phylogenies based on the 16S rRNA gene alone (left), and 350 genes conserved across at least two genomes (right). Node numbers represent posterior probabilities of Bayesian analyses.

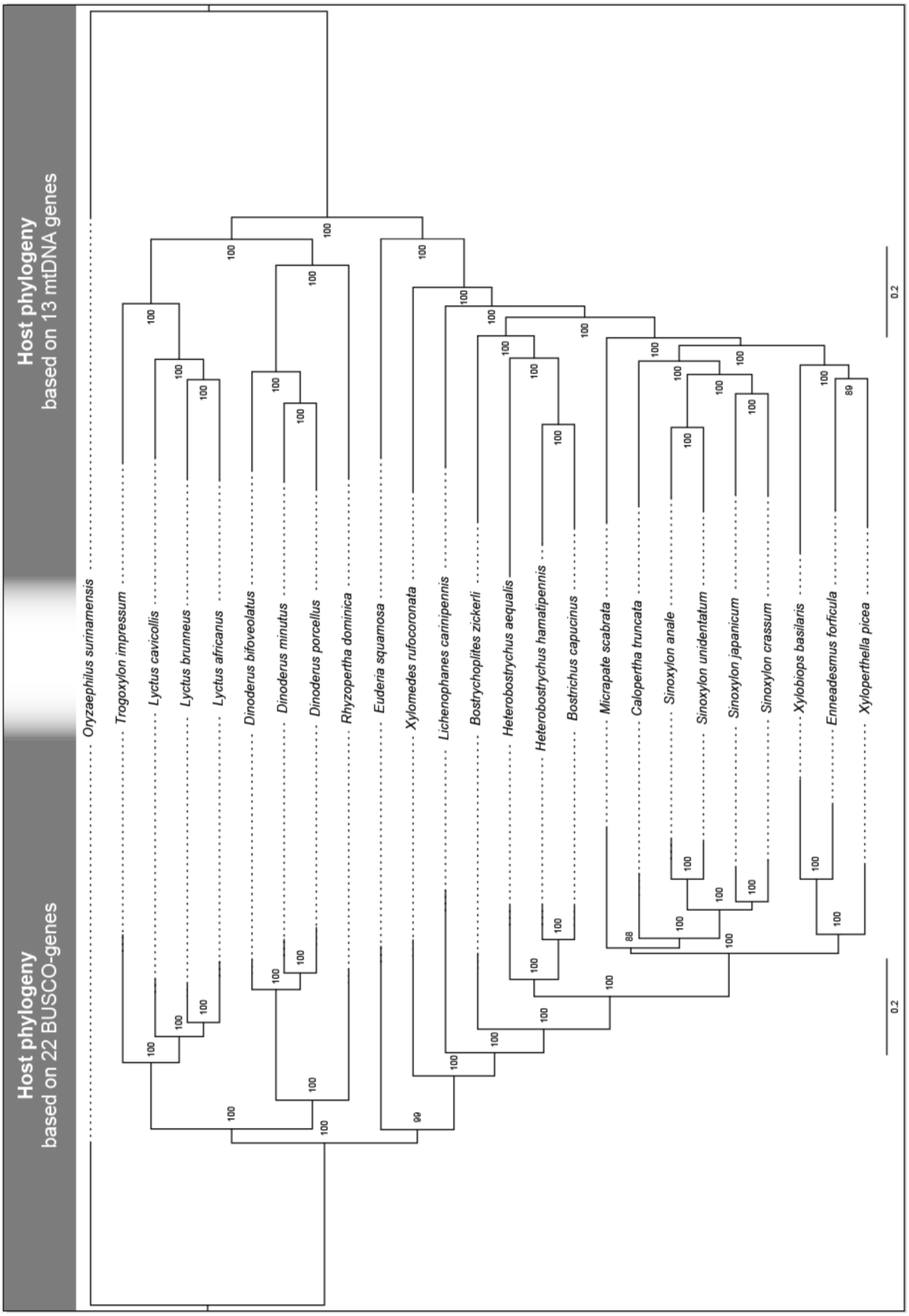

Figure S2: Comparison of host phylogenies based on 22 BUSCO genes (left) and 13 mitochondrial genes (right). Hosts with less than 22 annotated BUSCO genes were omitted from the analysis to achieve higher phylogenetic resolution. Node numbers represent posterior probabilities of Bayesian analyses.

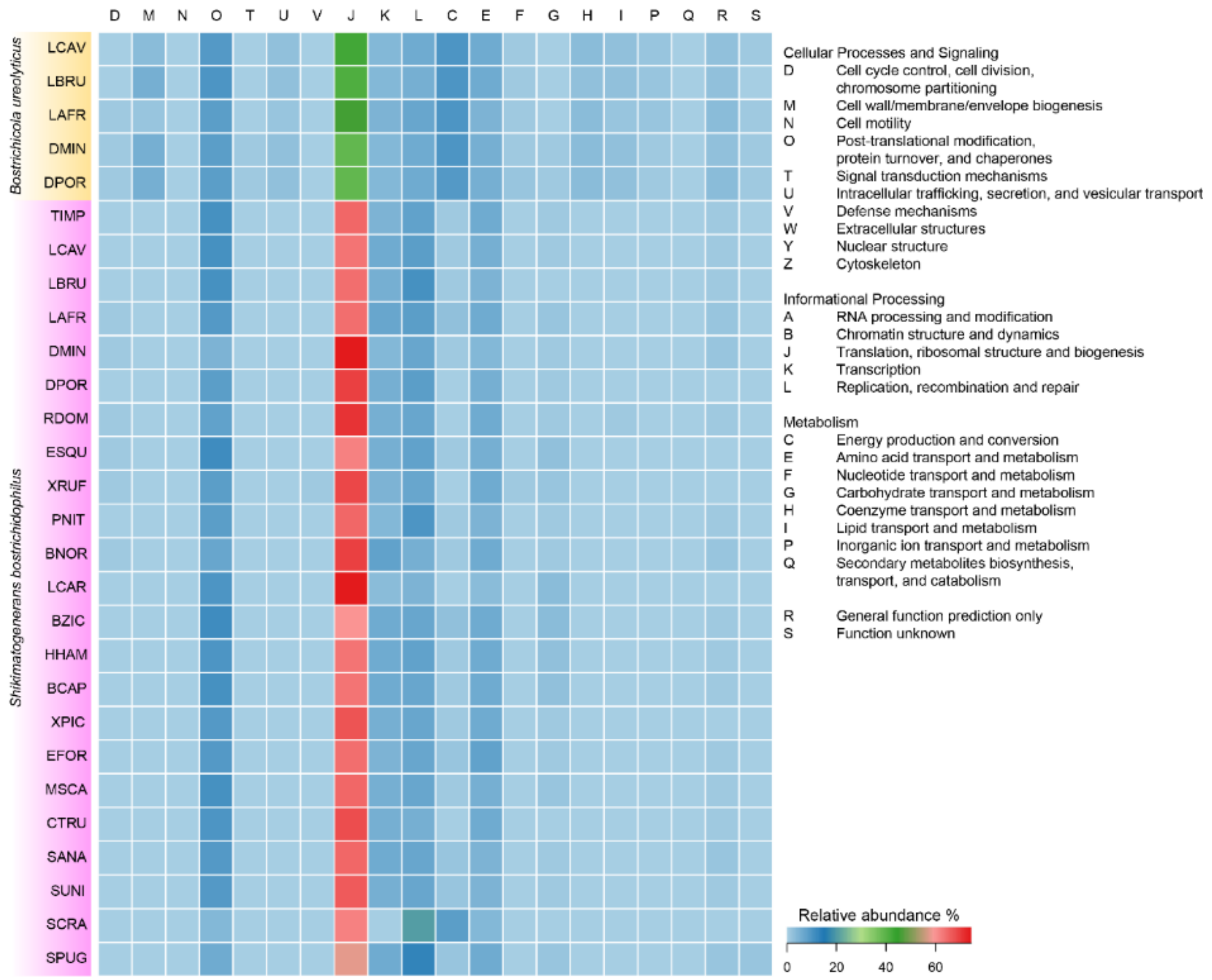

Figure S3: Relative abundance of Clusters of Orthologous Groups (COG). Annotated functional categories (A-Z) and relative proportion of the encoded genes represented as a heatmap are indicated on the right-hand side.

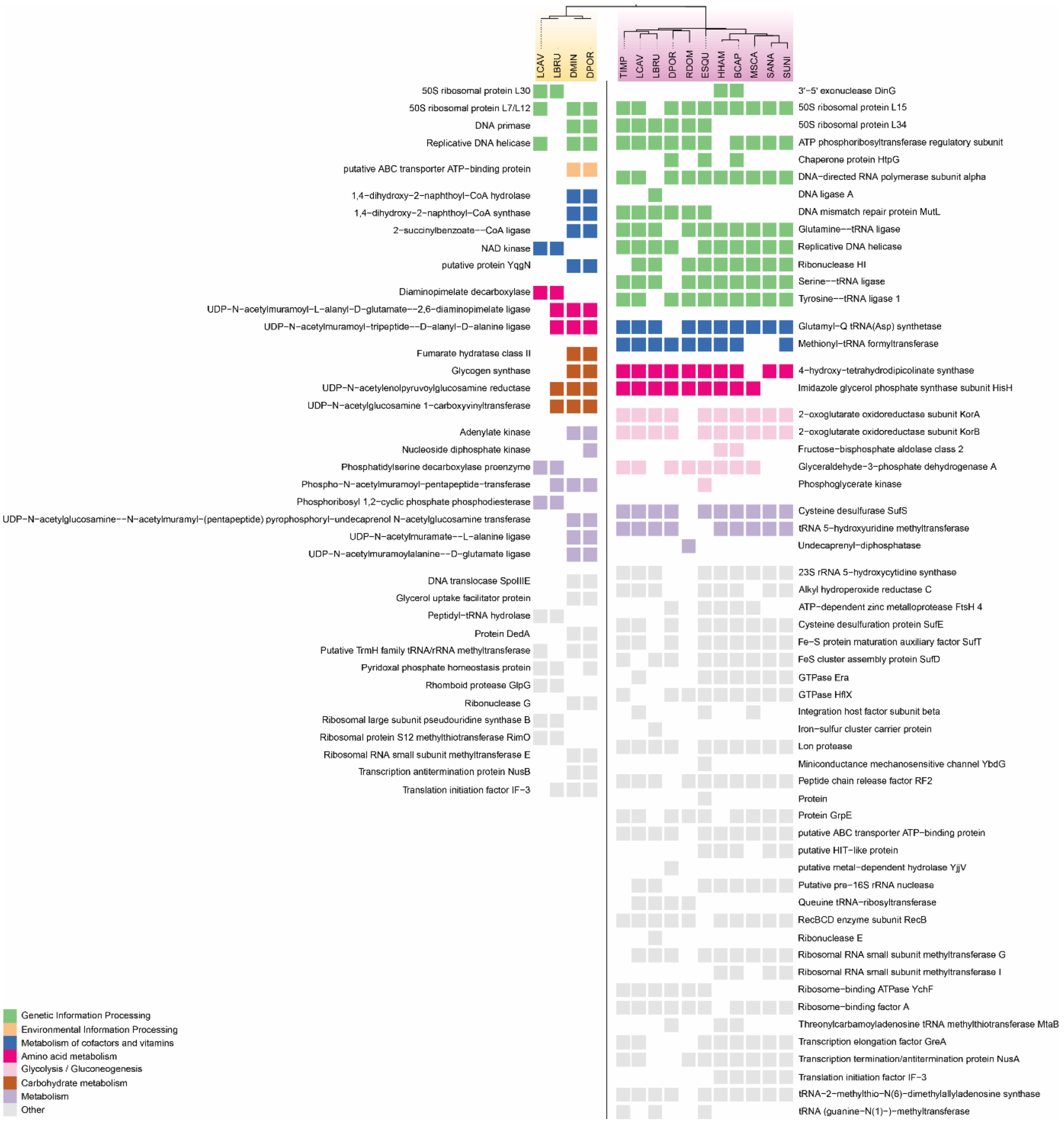

Figure S4: Comparison of all differentially encoded genes between genomes of *Bostrichicola ureolyticus* (left) and *Shikimatogenerans bostrichidophilus* (right). Genomes were annotated with PROKKA. Hypothetical proteins were removed. Filled: present, white: missing. Box colours are based on KEGG's categories (see legend for depicted categories)
